# Thermoresponsive gels containing gold nanoparticles as smart antibacterial and wound healing agents

**DOI:** 10.1038/s41598-018-31895-4

**Published:** 2018-09-12

**Authors:** Mona G. Arafa, Reham F. El-Kased, M. M. Elmazar

**Affiliations:** 1 Department of Pharmaceutics, Faculty of Pharmacy, The British University in Egypt (BUE), El-Sherouk City, Cairo 11837 Egypt; 2 Department of Microbiology & Immunology Faculty of Pharmacy, The British University in Egypt (BUE), El-Sherouk City, Cairo 11837 Egypt; 3 Department of Pharmacology, Faculty of Pharmacy, The British University in Egypt (BUE), El-Sherouk City, Cairo 11837 Egypt; 4 Chemotheraputic Unit, Mansoura University Hospitals, Mansoura, 35516 Egypt

## Abstract

Thermoresponsive gels containing gold nanoparticles (AuNPs) were prepared using Pluronic®127 alone (F1) and with hydroxypropyl methylcellulose (F2) at ratios of 15% w/w and 15:1% w/w, respectively. AuNPs were evaluated for particle size, zeta-potential, polydispersity index (PDI), morphology and XRD pattern. AuNP-containing thermoresponsive gels were investigated for their gelation temperature, gel strength, bio-adhesive force, viscosity, drug content, *in vitro* release and *ex-vivo* permeation, in addition to *in vitro* antibacterial activity against bacteria found in burn infections, *Staphylococcus aureus*. *In vivo* burn healing and antibacterial activities were also investigated and compared with those of a commercial product using burn-induced infected wounds in mice. Spherical AuNPs sized 28.9–37.65 nm displayed a surface plasmon resonance band at 522 nm, a PDI of 0.461, and a zeta potential of 34.8 mV with a negative surface charge. F1 and F2 showed gelation temperatures of 37.2 °C and 32.3 °C, bio-adhesive forces of 2.45 ± 0.52 and 4.76 ± 0.84 dyne/cm^2^, viscosities of 10,165 ± 1.54 and 14,213 ± 2.31 cP, and gel strengths between 7.4 and 10.3 sec, respectively. The *in vitro* release values of F1 and F2 were 100% and 98.03% after 6 h, with permeation flux values of (J_1_) 0.2974 ± 2.85 and (J_2_) 0.2649 ± 1.43 (µg/cm^2^·h), respectively. The formulations showed antibacterial activity with the highest values for wound healing properties, as shown *in vivo* and by histopathological studies. This study demonstrates that a smart AuNPs thermoresponsive gel was successful as an antibacterial and wound healing transdermal drug delivery system.

## Introduction

Skin burns are one of the major causes of morbidity and mortality in burn patients due to susceptibility to infection. The disruption of the epidermal barrier, combined with the denaturation of proteins and lipids, provides a fertile environment that is rich in bacterial nutrients for microbial growth, making it significantly prone to infection^1^. A long list of microorganisms (including species present in normal skin microflora) has been observed to colonize burn wounds, where the most abundant species is *Staphylococcus aureus* (6.5–37.6%)^2^. The widespread use of antibacterial agents for the treatment of skin infections, including burns, in hospitals has favoured the emergence of multidrug resistant bacteria, such as *Klebsiella sp*. and *Staphylococcus aureus*, which has resulted in serious opportunistic infections that are difficult to treat. The search for new antibacterial agent is therefore essential^3^.

Nanotechnology has acquired its importance by using nanomaterials, which have enormous biological applications in various medical fields, including biomedicine^4^, pharmacology, parasitology, nanopharmaceutics and drug delivery systems^5–9^.

Gold nanoparticles (AuNPs) demonstrate the greatest potential for use in biological applications because they are highly biocompatible and have an extensive array of medical applications. AuNPs have distinctive physicochemical properties, such as stability in biological media and the adsorption of near infrared light^10^. AuNPs display a unique and tuneable surface plasmon resonance (SPR) that can be used during photothermal therapy^11^, in addition to gene and drug delivery^12^. The antibacterial effect of AuNPs has recently been an interesting research area, which makes them suitable for the potential complementary use with antibiotics^13,14^. AuNPs exert their antibacterial activity through the formation of holes in the bacterial cell wall, which lead to cell death due to the loss of cell contents. Furthermore, AuNPs can inhibit the transcription process by binding to bacterial DNA and preventing the uncoiling of DNA during transcription and can inhibit multidrug-resistant uropathogens, such as *Escherichia coli*, *Enterobacter cloacae complex*, *Pseudomonas aeruginosa*, *Staphylococcus aureus*, and methicillin-resistant *Staphylococcus aureus* (MRSA)^15,16^.

Systemically administered antibiotics can encounter difficulties reaching damaged skin tissue due to compromised blood circulation, making them ineffective for reducing bacterial counts in granulation wounds^17^. The use of topically applied antibacterial products is therefore preferable, due to the controlled effects, low systemic toxicity, ease of application, patient compliance and the avoidance of the first-pass effect^18^.

Hydrogels are considered to be ideal topical delivery systems^19^, in which a liquid phase is constrained within a three dimensional cross-linked polymeric network capable of entrapping large amounts of water or biological fluids. This characteristic property is due to the presence of a large number of hydrophilic groups^19–21^. Hydrogels are preferred for the treatment of wounds because they have a tendency to hold a high water content that helps to supply dry wounds with the necessary fluids, a smooth consistency that is similar to normal tissue^21^, the ability to remove excessive amounts of discharge from wet wounds^22^, and biodegradable and biocompatible properties. Hydrogels can be further be classified into thermal, cryogel and complex coacervate gels^23^. Thermoreversible or thermoresponsive gels undergo a phase transition with temperature changes outside of a specified range, changing from a solid to a liquid, from a liquid to a solid, or swelling or shrinking a polymer network. The temperature range required for these changes is called the sol-gel transition temperature or critical transition temperature. Pluronic® F127 (PF127) belongs to a group of polymers that exhibits thermoreversible/thermoresponsive properties in aqueous solutions. PF127 consists of hydrophobic poly propylene oxide (PPO) blocks at the centre and hydrophilic polyethylene oxide (PEO) blocks on either side, producing the tri-block structure of PEO-PPO-PEO^24^. PF127 is used in concentrations ranging from 10–30% as a controlled drug delivery system with gelling properties, and it also has a high solubilizing ability with negligible toxic effects^25–27^. To obtain a suitable gel strength using a minimal amount of PF127 and to improve the bioavailability and drug release rate, different additives, such as hydroxypropyl methylcellulose (HPMC), may be included to achieve a controlled drug release rate^28,29^. HPMC acts as a thickening agent that decreases the critical micellization value of PF127 and increases its viscosity^30,31^. The optimum ratio of PF127:HPMC concentrations has been reported as 15% (w/w):0.92% (w/w)^32^.

The aim of the present study is to prepare and characterize thermoresponsive gels containing AuNPs in PF127 (AuNPs-PF127) and in PF127 with HPMC (AuNPs-PF127-HPMC), to investigate the efficacy of gold nanoparticle containing thermoresponsive gels against the gram-positive bacteria *Staphylococcus aureus*, and to demonstrate antibacterial and wound healing effects of these gels.

## Methods

### Instruments

HAAKE RV3 viscometer with a digital circulating water bath (Goerzallee 249, Germany), digital precise shaking water bath (WSB-18, Dahan Scientific Co. Ltd., Korea), ultraviolet-visible spectrophotometer (V-630, Jasco, Japan), Elmasonic (S60 H, Elma Hans Schmidbauer GmbH, Germany), pH meter (CA 92634, Beckman Instruments Fullerton, USA), transmission electron microscope (JEOL JSM-6510 LV., Tokyo, Japan), X-ray diffractometer (D8, Bruker Co., Germany) and Zetasizer Nano-Zs90 (Malvern Instruments Ltd. MPT-Z, UK) were used.

### Materials and reagents

Citrate-capped gold nanoparticles were purchased from NanoTech Egypt for Photo Electronics, Egypt. Pluronic^®^F127 (PF127), purified poly(ethylene glycol)-*block*-poly(propylene glycol)-*block*-poly(ethylene glycol), CAS Number: 9003–11–6, was purchased from Sigma-Aldrich, Germany. Hydroxypropyl methylcellulose (HPMC) was kindly supplied by Cairo Pharmaceuticals Co., Egypt. Monopotassium dihydrogen phosphate and anhydrous dibasic sodium phosphate were purchased from Adwic, Al Nasr Pharmaceutical Chemicals Co. Egypt. Spectra/por dialysis cellophane membrane 12,000–14,000 Mwt cut off was used.

### Preparation of gold nanoparticles (AuNPs)

AuNPs were prepared using the Turkvich^33^ reduction process, in which a hydrogen tetrachloroaurate solution was used as a Au^3+^ ion precursor in the presence of sodium citrate as a reducing and stabilizing agent. The appearance of a faint pink colour in the final solution indicated the reduction of Au^3+^ ions to AuNPs.

#### Preparation of the AuNPs- PF127 thermoresponsive gel

Pluronic®F127 (PF127) is characterized by a thermoreversible gelling property; therefore, the cold method was implemented to prepare the gel^26^. Briefly, a defined volume of PF127 was slowly added to half the desired final volume of cold distilled water at 4 °C with constant stirring. A solution containing the desired amount of drug (5 × 10^-4^%w/w) was mixed with the polymeric solution under vigorous stirring to obtain a clear mixture, and then, the solution was adjusted to the final desired volume. The mixture was stored overnight at 4 °C until a glutinous and lucid translucent gel was obtained (Table 1).

**Table 1:** Compositions of different thermoresponsive gels.

Formua	Ingredients			
	HPMC (g)	PF127 (g)	Gold nanoparticles (mg)	Distilled water (g)
F1	—	15	0.5	100
F2	1	15	0.5	100

#### Preparation of the AuNps- PF127- HPMC thermoresponsive gel

A defined volume of PF127 was added to half the desired final volume of distilled water containing an accurately weighed amount of HPMC. Preparation then continued using the cold method, as previously described^26^ (Table 1).

### Characterization of AuNPs

#### UV–visible spectroscopy

The UV-vis spectrum of Au sol was determined spectrophotometrically (Jasco, UK model no. V-630) at wave lengths of 200–800 nm. To obtain the spectra of localized surface plasmon resonance (LSPR) generated by AuNPs, quartz crystal cuvettes containing 2 ml of 1% wt. sodium citrate were used to obtain a baseline measurement. Au sol (2 ml) was analysed using scan speeds of 200 nm/min to record the proper wavelength.

#### Transmission electron microscopy (TEM) analysis of AuNPs

Copper grids were prepared by placing 100 µl of 1.2 nM Au sol on the surface and allowed to settle for 2 min before removing the excess liquid with filter paper. The grids were air dried before being analysed with the electron microscope (JEOL JSM-6510 LV., Tokyo, Japan)^34^.

#### X-ray diffraction (XRD) analysis of AuNPs

The synthesized gold nanoparticles were examined for structure and phase purity by X-ray diffraction (XRD) analysis^35^ using the Turkevich method.

#### Determination of particle sizes, size distributions and zeta potentials of AuNPs

Measuring the zeta potential of the particles is essential for determining their surface charge. In addition to determining the particle sizes, zeta potential was measured using a Zetasizer Nano-Zs90 (Malvern International Ltd, MPT-Z, UK). The size distribution investigation was performed using the tested samples at a scattering angle of 90 degrees and at 25 °C. For each sample, the mean diameter ± SD of 10 runs was calculated by applying multimodal examination^36^.

### Evaluation of AuNPs thermoresponsive gels

#### Determination of AuNPs contents in the thermoresponsive gels

All prepared gels were analysed for AuNPs content, with a desired range of 100 ± 10%^37^. Briefly, 100 mg of each thermoresponsive gel was added to 10 ml of distilled water and mixed for 15 min. After filtration, the filtrate was suitably diluted with distilled water and the absorbance of the solution was measured at 522 nm spectrophotometrically. The experiments were performed in triplicate.

#### Measurement of the gelation temperatures of the AuNPs thermoresponsive gels

A 2 ml aliquot of each thermoresponsive gel was placed in a test tube that was wrapped with aluminium foil and submerged in a water bath at 4 °C. The temperature of the water bath was raised by 1 °C every 3–5 minutes until the gelation temperature was reached. The temperature was raised continually until no mobilization of the gel occurred upon tilting the tube through 90 degrees. The minimal temperature at which immobility was attained for each tube was considered to be the sol-gel transition temperature for that concentration^38^.

#### Measurement of the gel strengths of the AuNPs thermoresponsive gels

A weighed amount of each medicated gel was placed in a 100 ml graduated cylinder and gelled at 32–37 °C, corresponding to their individual gelation temperatures. A 35 g weight was placed on each gel^28^. The gel strength was specified as the time (in sec) required for the weight to descend 5 cm.

#### Determination of the bioadhesive forces of the AuNPs thermoresponsive gels

The bio-adhesive forces of the thermoresponsive gels was determined using the procedure described by Choi *et al*.^39^ and a technique modified by Hongyi *et al*.^40^. Using two glass vials, a piece of skin was excised from the dorsal region a mouse and immediately placed onto the vials using a rubber crew with its mucosal side out and maintained at 37 °C. One vial (V1) was attached to the right side of the balance in an overturned pose, while the other vial (V2) was located on the right balance pan (P1) in the upright position. The tested gel was added onto the skin tissue on both vials, and then, the height of both vials was adjusted until they were connected by the thermoresponsive gel. Water from a burette was allowed to fall at a constant rate of 10 mg/sec into a beaker placed on the left balance pan (P2). The weight of the added water was gradually increased until the two vials detached^39^. The minimum weight that detached the two vials was used in the following (Equation 1), reported by Ch’ng *et al*.^41^, to calculate the bio-adhesive force:

1 $$F=\frac{({\rm{Ww}}\times {\rm{g}})}{{\rm{A}}}\,$$

where

F = The bio-adhesive force (dyne/cm^2^);

Ww = The weight of the water (g);

g = The acceleration due to gravity (cm/sec^2^); and

A = The surface area of the mucous membrane (cm^2^).

The experiment was performed in triplicate for each formulation and the mean bio-adhesive force was determined.

#### Determination of the viscosities of the AuNPs thermoresponsive gels

The rheological evaluation of the prepared thermoresponsive gels was determined using a rotary viscometer (con and plate viscometer), according to the method by Liu *et al*.^42^. One gram of each gel was spread on the 2.9 cm diameter plate of the viscometer and on the 2.8 cm diameter Roto Cone. The viscosity of each sample was measured at a 32 rpm angular velocity and at a temperature of 32–37 ± 0.5 °C, corresponding to the gelation temperature of each sample. The angular velocity was reversed, and the operation was repeated twice to compute the velocity. The viscosity was determined using (Equation 2) from the instrumental manuscript:

2 $${\rm{\eta }}=\frac{{\rm{G}}{\rm{.S}}}{{\rm{N}}}$$

where η is the viscosity of the thermoresponsive gels in mPa·s, G is the instrumental factor that equals 14,200 (mPa·s/scale grade·min.), S is the torque (scale grade), and N is the rotating speed (rpm).

### *In vitro* release study of AuNPs from the thermoresponsive gels

The diffusion cells were thermo-regulated using a water bath at 37 ± 0.5 °C. The semi-permeable cellophane membranes were immersed in phosphate buffer (pH 7.4) and then mounted using a rubber band to cover the open end of a glass tube with a 3 cm diameter and a 7.068 cm^2^ diffusion area.

One gram of each formula (equivalent to 5 µg of AuNPs) was thoroughly distributed on the membrane to cover the 3 cm diameter, and 1.5 ml of the same solution was added to every tube. The tubes were then inverted and submerged in a beaker containing 50 mL phosphate buffer (pH 7.4) and maintained at 37 ± 0.5 °C. The whole assembly was shaken at 25 strokes per minute for the duration of the diffusion protocol. For each gel sample, and at predetermined time points of 1, 2, 3, 4, 5, 6, 7 and 8 h, aliquots were withdrawn and replaced with equal volumes of freshly prepared medium maintained at the same temperature. The released amounts of AuNPs were analysed spectrophotometrically at 522 nm.

### *Ex vivo* permeation study of the AuNPs thermoresponsive gels

The *ex vivo* permeation of AuNPs from the transdermal thermoresponsive gels, F1 and F2, was measured using excised dorsal skin from mice. All animal work was conducted in accordance with the guidelines outlined in the *Guide for the Care and Use of Laboratory Animals* and was approved by the Ethical Committee of Faculty of Pharmacy, The British University in Egypt^18^. The *ex vivo* permeation was performed using Franz diffusion cells. The prepared dorsal skin was placed between the two chambers of the cell and a stratum corneum was fixed to face the donor chamber. Receptor units contained phosphate buffer (pH 7.4) and magnetic stirrer bars^18^. The whole system was positioned on a magnetic stirrer and was maintained at 37 ± 0.5 °C. One gram of each prepared transdermal thermoresponsive gel containing 5 µg AuNPs was applied to the prepared skin. Samples were collected from the receptor chambers at specified time points, with the addition of fresh receptor liquid to maintain the skin conditions. The amount of permeated AuNPs was determined spectrophotometrically at 522 nm.

The permeation flux was calculated^43^ using (Equation 3)

3 $${\rm{Jss}}=\mathrm{dQ}/({\rm{dt}}\,\ast \,{\rm{A}})$$

where:

Jss = steady-state permeation flux (µg/cm^2^/h);

A (cm^2^) = the area of the skin tissue that the drug permeated over time t; and

(dQ/dt) = the amount of drug passing *via* skin/unit time at a steady state.

### Kinetic study

*In vitro* release results were investigated using linear regression, according to zero-order, first-order and Higuchi diffusion models^18^, to understand the AuNPs release profiles from PF127 and PF127-HPMC systems. In addition, the Korsmeyer–Peppas model was determined using (Equation 4)^44^.

4 $$({\rm{Mt}}/{\rm{M}}\infty ={\rm{ktn}})$$

where:

Mt = the amount of drug released at time t;

M∞ = the amount of drug released as time approaches infinity;

k = a constant incorporating characteristics of the particle system or network; and

n = the diffusion exponent.

### *In vitro* antibacterial evaluation of AuNPs

The agar well diffusion method was used to evaluate the antibacterial activity of an AuNPs stock solution (200 µg/mL). Three different concentrations of the AuNPs stock solution were investigated, containing 0.25, 0.5 and 2.5 µg AuNPs. The test was performed against *Staphylococcus aureus*, the strain most commonly isolated from burn infections. Mueller Hinton agar (Oxoid, UK) was used, and the agar surface was inoculated by spreading a standard suspension of *Staphylococcus aureus* inoculum (adjusted to a density of 1.5 × 10^8^ CFU/mL in 0.5 McFarland reagent) over the entire surface using a sterile spreader. Dishes were allowed to stand for 10 min to dry. A hole with a diameter of 6 mm was punched aseptically with a sterile cork borer, and then, the AuNPs solutions were added to the well using a micropipette. Agar plates were incubated at 37 °C for 24 h, and zones of inhibition were measured.

### *In vitro* antibacterial examination of the AuNPs thermoresponsive gels

For the evaluation of the antibacterial properties of the prepared gels (F1 and F2), the disk diffusion antibiotic sensitivity test (Kirby-Bauer) was used^45^. The test was employed against the bacterial strain most commonly isolated from burn infections, *Staphylococcus aureus*. Mueller Hinton agar (Oxoid, UK) was used. Manufacturer’s instructions were followed during the preparation of all media. The method described by Koneman *et al*.^46^ was used to prepare 5 mL of 0.5 McFarland standard in a sterile test tube. From the *Staphylococcus aureus* bacterial suspension, an inoculum was prepared. Briefly, 5–6 bacterial isolate colonies were emulsified in sterile distilled water, and the turbidity was adjusted to 1.5 × 10^8^ CFU/mL (equivalent to 0.5 McFarland standard). Afterwards, a sterile cotton swab was immersed into the standardized bacterial suspension and inoculated evenly on the agar plates. Plates were left to dry for 5 minutes. Filter paper disks (6 mm diameter) were prepared according to Cheesbrough^47^. Disks were soaked in the two prepared formulations (F1 and F2) until saturation was reached. The formulation-impregnated disks were placed on the inoculated agar plates and forced lightly to ensure full contact with the agar. To prevent the intersection of inhibition zones a space of 20 mm was maintained from the edges of the plates. An oxacillin disk (5 µg) and silver sulphadiazine cream were used as positive controls. After 15 minutes, all plates were incubated at 37 °C for 24 h. The plates were inspected and the diameters of the zones of inhibition were measured.

### *In vivo* antibacterial assessment and burn healing evaluation of *AuNPs thermoresponsive gels*

#### Burn induction and the establishment of burn wound infections

For the evaluation of the burn healing properties of the prepared formulations, an animal model of burn wounds was used. A total of 10 albino mice (weight 30–35 g), approximately 8 weeks old, were used. Animals were individually maintained in clean polyethylene cages under standard experimental conditions at a temperature of 23 ± 2 °C. Anaesthetic ether was used to anaesthetize the mice, and then, the backs of the mice were shaved first with an electric clipper then with a razor and shaving cream. The shaved area was disinfected with 70% (v/v) ethanol. The burn wounds were induced as described by Gurung and Skalko-Basnet^48^. Briefly, a cylindrical metal rod (10 mm diameter) was heated over an open flame for 20 seconds and then pressed to the shaved and disinfected dorsal mouse skin surface for 10 seconds under light ether anaesthesia, forming a burn wound 10 mm in diameter. Four burn wounds were induced on the back of each mouse (two on each side).

The burn wounds on all mice were inoculated with 1.5 × 10^7^ colony forming unit (CFU) of *Staphylococcus aureus*. Inoculation was performed by rubbing a 1 mL bacteria suspension directly onto the fresh wound using a sterile cotton swab. The inoculum was spread evenly across the wound surface.

The experimental protocol was approved by the Animal Care and Use Committee of The British University in Egypt, and the study was performed according to the animal welfare guidelines and regulations.

#### The effects of the AuNPs thermoresponsive gels on the inhibition of wound area

The size of the wound diameter was the primary criterion for indicating progressive healing, where a burn edge contraction was expressed as a decrease from the original burn diameter. Each mouse received four treatments, one treatment per wound area, such that one wound per mouse received each treatment: formula 1 (F1), formula 2 (F2), a standard wound healing cream (silver sulphadiazine) as a positive control (P), and normal saline as a negative control (N). Applications were used in a rotational manner (F1F2PN, F2PNF1, PNF1F2, and F2PNF1) between mice^49^. Using a sterile cotton swab, all treatments (100 mg) were applied topically to each mouse once daily and were continued until the apparent skin healing due to any of the applications. The mice were fed on commercial pellets and water *ad libitum* throughout the study.

A digital calliper was used to measure the changes in burn edge diameter (mm) every day prior to treatment applications. All measurements were documented for further analysis.

#### Bacterial infection assessment of the induced burns

*In vivo* antibacterial effects of the AuNPs gels: Surface swabs from burn wound positions were collected daily prior to the applications of the topical treatments to evaluate the antibacterial activities of the tested formulations in comparison with the positive control, the commercial product silver sulphadiazine. A sterile cotton swab was used to swab the total burned area, and the swabs were rotated over the wound area for five seconds with appropriate pressure. Swab samples were collected from the burn wound areas with the maximal degree of burn. The samples were homogenized in 2 mL sterile saline, and then, successive dilutions were made and plated on sterile agar media. All plates were incubated under aerobic conditions at 37 °C for 24 h. Afterwards, bacterial colonies were counted on each plate by visual inspection. Colony forming units were then used to determine the total bacterial count on each plate.

### Histopathological analysis

Autopsy samples were taken from the skin of each mouse after apparent skin healing was reached. Four samples were taken from each mouse, representing the locations of the treatments, for a total of 40 samples, 10 for each treatment. Mice received brief ether anaesthesia followed by cervical dislocation, after which skin samples were removed and fixed in a 10% formalin solution for 24 h. Samples were then washed with tap water, followed by dehydration using serial dilutions of alcohol (methyl alcohol, ethyl alcohol and absolute ethyl alcohol). Afterwards, the specimens were cleared using Xylene and fixed in paraffin at 56 °C in a hot air oven for 24 h. Tissue blocks in paraffin beeswax were prepared for sectioning at a thickness of 4 microns using a sledge microtome. Prepared tissue sections were placed on glass slides, de-paraffinized and stained with haematoxylin and eosin stain (H and E) for examination using an electric light microscope^50^. Digital photomicrographs were taken at representative sites, and the burns were evaluated for the extent of acanthosis, new blood capillary formation and regeneration.

### Skin irritation test

Rabbits were prepared by removing the fur from a section of the back. F1 and F2 (0.2 g) were applied topically to the skin and maintained in contact for 4 h. The degree of skin irritation was scored for erythema, eschar and oedema formation and corrosive action. The observations were repeated at various intervals for 24 h.

### Skin sensitization experiments

Guinea pigs were prepared by removing the fur from a section of the back. F1 and F2 (0.2 g) were applied topically to the skin daily for a period of two weeks. Two weeks after the last application, the animals were challenged by an application of 0.2 g of F1 and F2 and the development of an erythaematous response was evaluated.

### Statistical analysis

Statistical analysis was performed using an ordinary non-linear regression and Student’s paired t-test. Significance was achieved at a p value < 0.05. The tests were performed using the Prism 5 computer program (Graph Pad software Inc., 5 Demo. Ink.).

### Ethics Approval

All animal work was conducted in accordance with the guidelines outlined in the *Guide for the Care and Use of Laboratory Animals* and was approved by the Ethical Committee of Faculty of Pharmacy, The British University in Egypt.

## Results

### Characterization of the AuNPs

**Figure 1:**
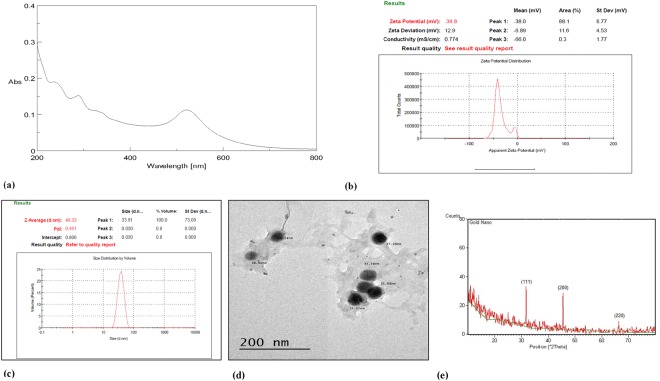
The UV spectrum (**a**) zeta potential (**b**) particle size and particle size distribution (**c**) transmission electron microscopy (**d**) and X-ray diffraction (**e**) of formed gold nanoparticles (AuNPs).

The recorded UV spectrum in the present study showed a well-defined surface plasmon resonance (SPR) band at 522 nm, which is the characteristic absorbance wavelength of AuNPs^51^ (Fig. 1a). The zeta potential of the AuNPs showed a negative surface charge and the mean zeta potential equalled (−34.8 mV) (Fig. 1b). The present results match those reported by Xiao *et al*.^52^ and exceed those reported by Khademi *et al*.^53^

The size of the AuNPs was found to be 33.81 nm, with a polydispersity index (PDI) of 0.461 (Fig. 1c).

TEM analysis was performed to comprehend the shapes and sizes of the AuNPs, and the results showed the formation of triangular to spherical nanoparticles 28.9–37.65 nm in size, with no aggregation (Fig. 1d). The XRD patterns of the AuNPs were recorded in a spectrum of 2θ from 10° to 70°, and the results showed the presence of the three characteristic intense peaks of 31.7°, 45.4° and 67.7°, corresponding to the (111), (200) and (220) planes of the face-centred cubic AuNPs. These results are in accordance with many reports^35,54–56^ and confirm the formation of crystalline, non-aggregated AuNPs^56^ (Fig. 1e).

### Evaluation of the AuNPs thermoresponsive gels

#### Measurement of the gelation temperatures of the AuNPs thermoresponsive gels

**Figure 2:**
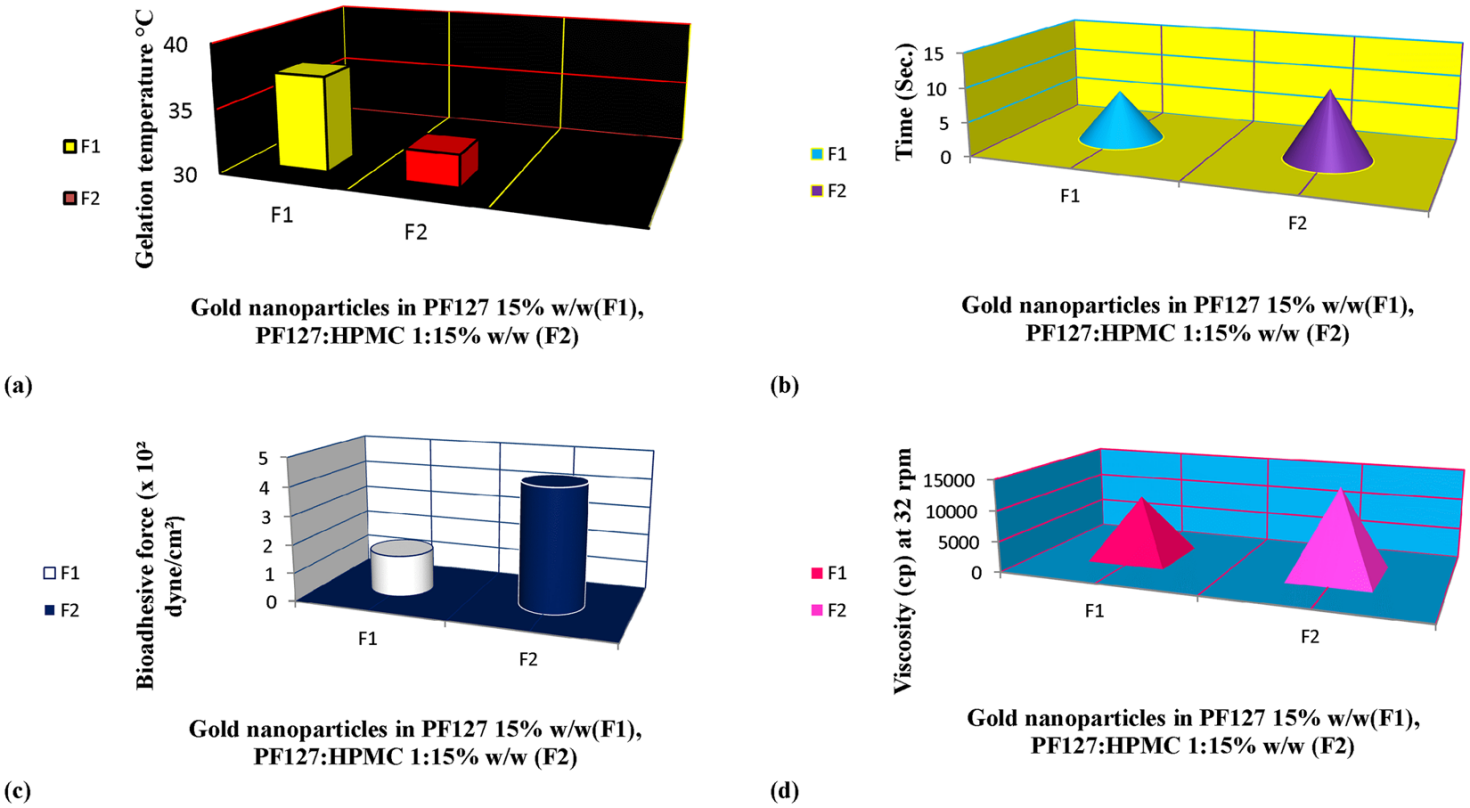
Gelation temperature (**a**) gel strength (**b**) bioadhesive force (**c**) and viscosity profile at 32 rpm (**d**) of F1 (AuNPs-PF127) and F2 (AuNPs-PF127-HPMC) thermoresponsive gels.

When F1 and F2 were maintained at 4 °C, the two formulations existed in liquid form. The results in Table 2 show that F1 and F2 exhibited gelling temperatures of 37.23 °C and 32.37 °C, respectively, as determined by increasing the temperature gradually. In the present experiment, the PF127 formula existed as a soft gel at 37 °C and became a stiff gel at a lower temperature when HPMC was added to the PF127 matrix (Fig. 2a).

**Table 2:** Physicochemical properties of the different AuNPs thermoresponsive gels at 37 °C.

Formula	Rheological Properties			
	Viscosity cP at 32 rpm	Rheometry gel strength (sec)	Bio-adhesive force (dyne/cm^2^)	Gelling temperature (°C)
F1	10,165 ± 1.54	7.4 ± 0.33	2.45 ± 0.52	37.23 ± 0.33
F2	14,213 ± 2.31	10.3 ± 0.45	4.76 ± 0.84	32.37 ± 0.25

#### Measurement of the gel strengths of the AuNPs thermoresponsive gels

The results showed that F1 and F2 exhibited gel strengths of 7.4 and 10.3 sec and that shear thinning occurred between 32.37 °C and 37.23 °C, respectively (Table 2). As shown in (Fig. 2a), the gelation temperature is the temperature at which an increment of gel strength was detected. The gelation temperature of PF127 decreased in the presence of HPMC, while the gel strength increased. The time necessary for the weight (35 g) to travel through F2 was longer than that for F1, demonstrating that the gel strength of F2 was increased (PF127:HPMC 15:1% w/w) compared to F1 (PF127 15% w/w alone).

#### Determination of the bio-adhesive forces of the AuNPs thermoresponsive gels

The results shown in (Table 2) and (Fig. 2c) illustrate that the bio-adhesive forces of F1 and F2 were 2.45 ± 0.52 dyne/cm^2^ and 4.76 ± 0.84 dyne/cm^2^, respectively.

#### Determination of the viscosities of the AuNPs thermoresponsive gels

The viscosities of F1 and F2 were found to be in the range of 10165 ± 1.54 to 14213 ± 2.31 cP, respectively, for 10 runs at 32 rpm (Table 2). The viscosity of PF127 15% w/w increased in the presence of HPMC at 1% (w/w) (Fig. 2d).

### *In vitro* release of AuNPs from the thermoresponsive gels

**Figure 3:**
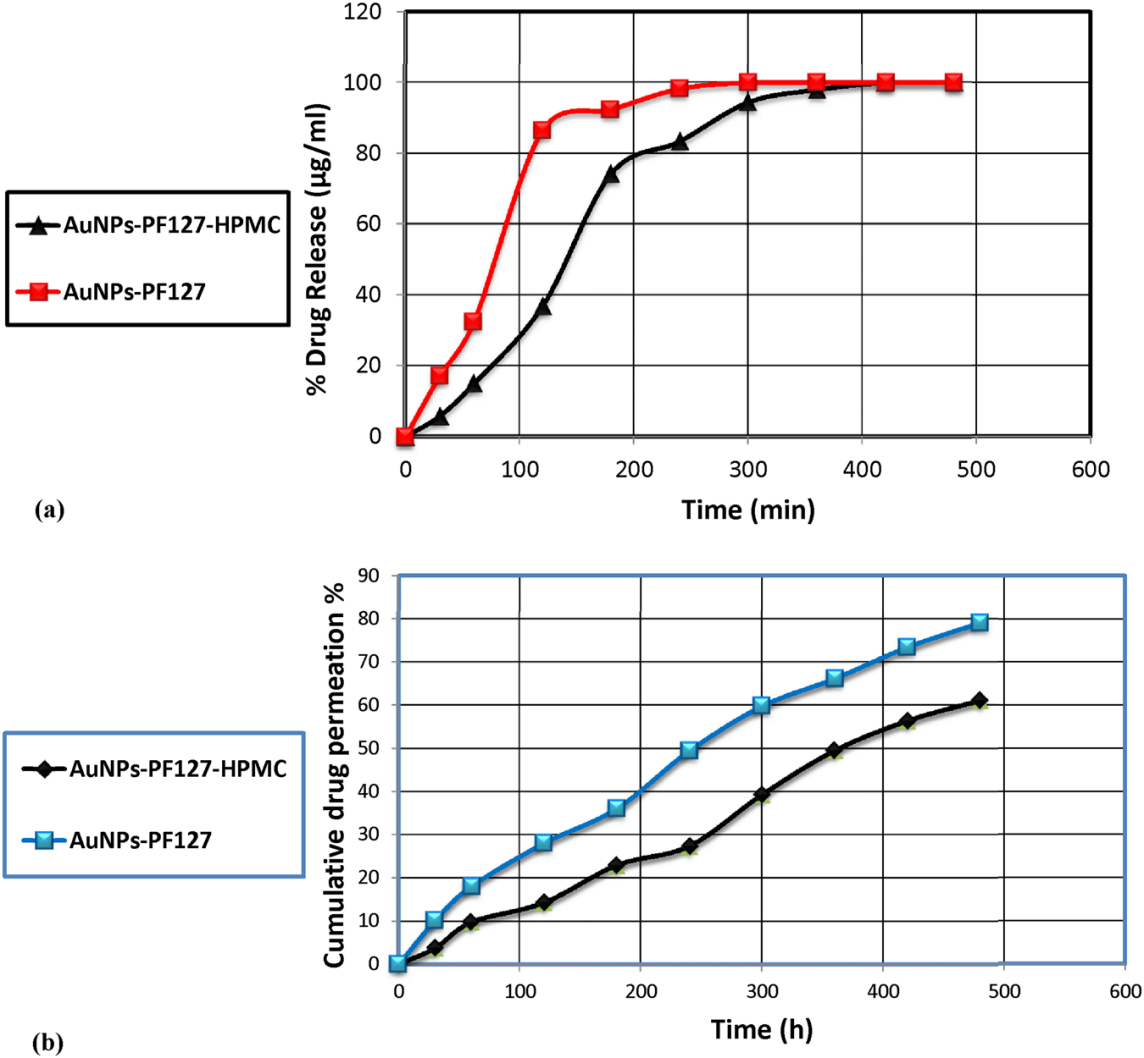
Release of AuNPs from PF127 and PF127-HPMC thermoresponsive gels in phosphate buffer (pH 7.4) at 37 °C. (**a**) and Permeation studies through excised mice skin by F1(AuNPs-PF127) and F2 (AuNPs-PF127-HPMC) thermoresponsive gels (**b**) with the same drug concentration (0.5% w/w).

The release of the drug was found to be dependent on the gelling agent PF127 and the drug release modifier HPMC. The results showed that 100% and 98.03% of AuNPs were released from F1 and F2, respectively, 6 h (Fig. 3a). Because the F1 and F2 formulations showed gelation temperatures of 32 ± 0.37 °C and 37 ± 0.23 °C and optimum viscosities of 10,165 ± 1.54 and 14,213 ± 2.31 cP, respectively, and released the desired drug for up to 6 h, F1 and F2 were used for further *ex vivo* and *in vivo* investigations.

### *Ex vivo* permeation study of the AuNPs thermoresponsive gels

F1 and F2 showed 49.405 ± 1.84% and 27.37 ± 2.13% drug permeation over 5 h, respectively, with permeation flux values of (J) of 0.2974 ± 2.85 (µg/cm^2^·h) and 0.2649 ± 1.43 (µg/cm^2^·h), respectively, (Fig. 3b). The *ex vivo* permeation study of the two formulations showed low flux values and sustained release profiles, indicating the continued presence of AuNPs at the site of action for up to 5 h, with no significant difference (p > 0.05) between F1 and F2.

### Kinetic study

The results revealed that the *in vitro* release of AuNPs from the PF127 gel formulation could be explained by zero order kinetics at 32–37 ± 0.5 °C, as the plots showed the maximum linearity (R^2^ = 0.973) (Table 3). Meanwhile, the *in vitro* release of AuNPs from the PF127-HPMC gel was best explained by first order kinetics at 32–37 ± 0.5 °C, as the plots showed the primary linearity (R^2^ = 0.849)^57^.

**Table 3:** The kinetic release profiles of the gel systems for AuNPs.

Correlation coefficient r^2^			Korsmeyer-Peppas model			Main Transport mechanism
Formula	Zero Order	First order	r^2^	n	k	
**F1**	0.973	—	0.945	0.744	0.326	Non-Fickian
**F2**	—	0.849	0.854	0.689	0.164	Non-Fickian

All data were also fitted to the Korsmeyer–Peppas Equation. A more acceptable linearity was obtained, and the values of the release exponent ‘n’ were 0.744 and 0.689 for F1 and F2, respectively. This result revealed that the drug release was controlled by non-Fickian or anomalous transport, indicating that the dissolution of the gel sustained the AuNPs release^58^.

### *In vitro* antibacterial evaluation of AuNPs

**Figure 4:**
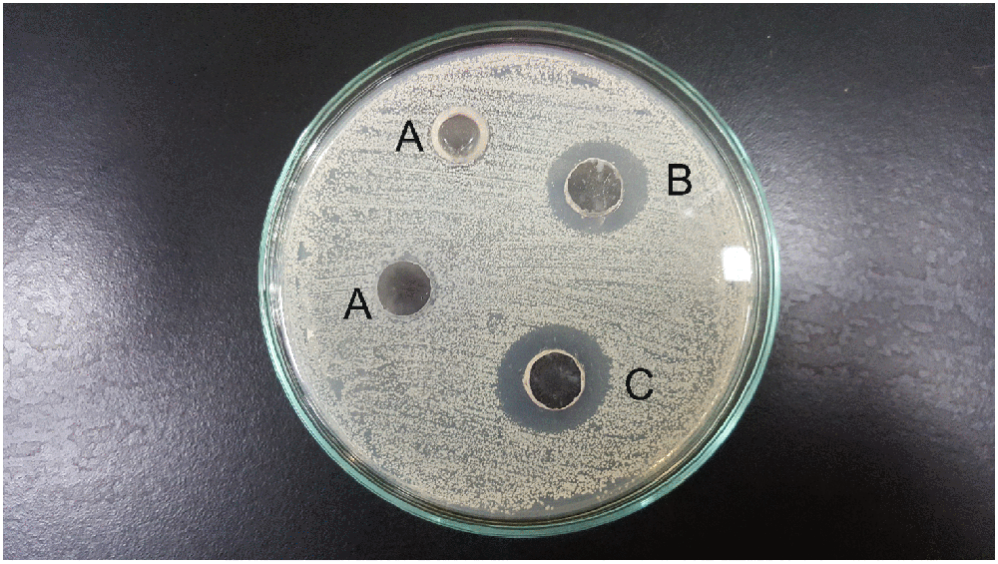
Antibacterial activity of gold stock solution against *Staphylococcus aureus*, determined by agar well diffusion method. A: 0.25 µg Au (**a**), B: 0.5 µg Au (**b**) and C: 2.5 µg Au (**c**).

Three different concentrations of AuNPs stock solutions were investigated; 0.25, 0.5 and 2.5 µg (Fig. 4). The lowest concentration of AuNPs (0.25 µg) did not show any antibacterial activity, while the medium and highest concentrations (0.5 and 2.5 µg) presented nearly identical antibacterial activity, as estimated by the measured zones of inhibition (15 and 13 mm, respectively). Therefore, the lowest concentration that achieved the maximal antibacterial activity (0.5 µg) was chosen for further studies.

### *In vitro* antibacterial examination of the AuNPs thermoresponsive gels

**Figure 5:**
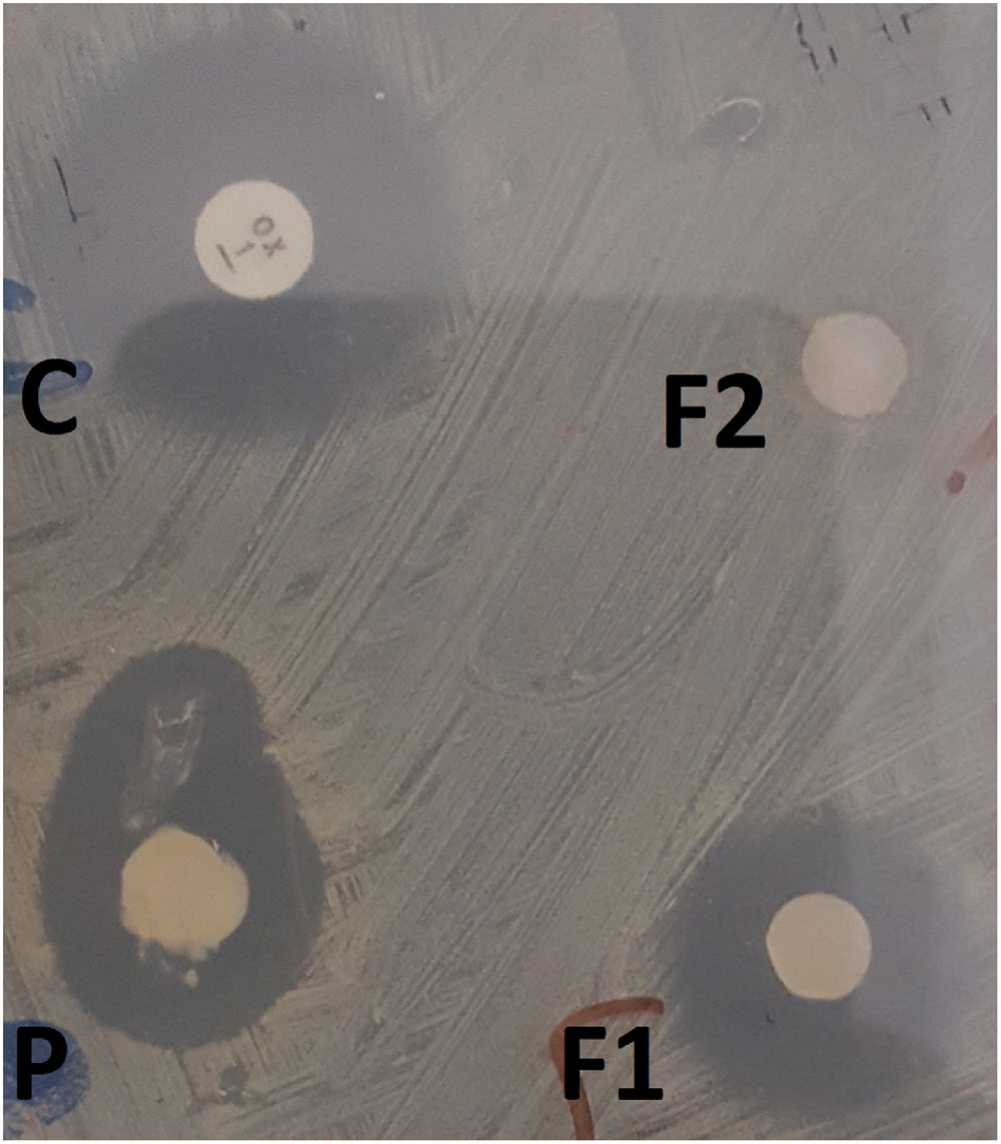
Antibacterial activity of the prepared formulations (F1 and F2) against *Staphylococcus aureus*, determined by disc diffusion antibiotic sensitivity method. C: control; Oxacillin antibiotic disc, P: commercial product; silver sulphadiazine, F1: (AuNPs-PF127) and F2: (AuNPs-PF127-HPMC).

F1 showed more rapid antibacterial activity when compared to F2 by measuring the zones of inhibition after 24 h (Fig. 5).

### *In vivo* antibacterial assessment and burn healing evaluation of the *AuNPs thermoresponsive gels*

#### Burn induction and the establishment of burn wound infections

**Figure 6:**
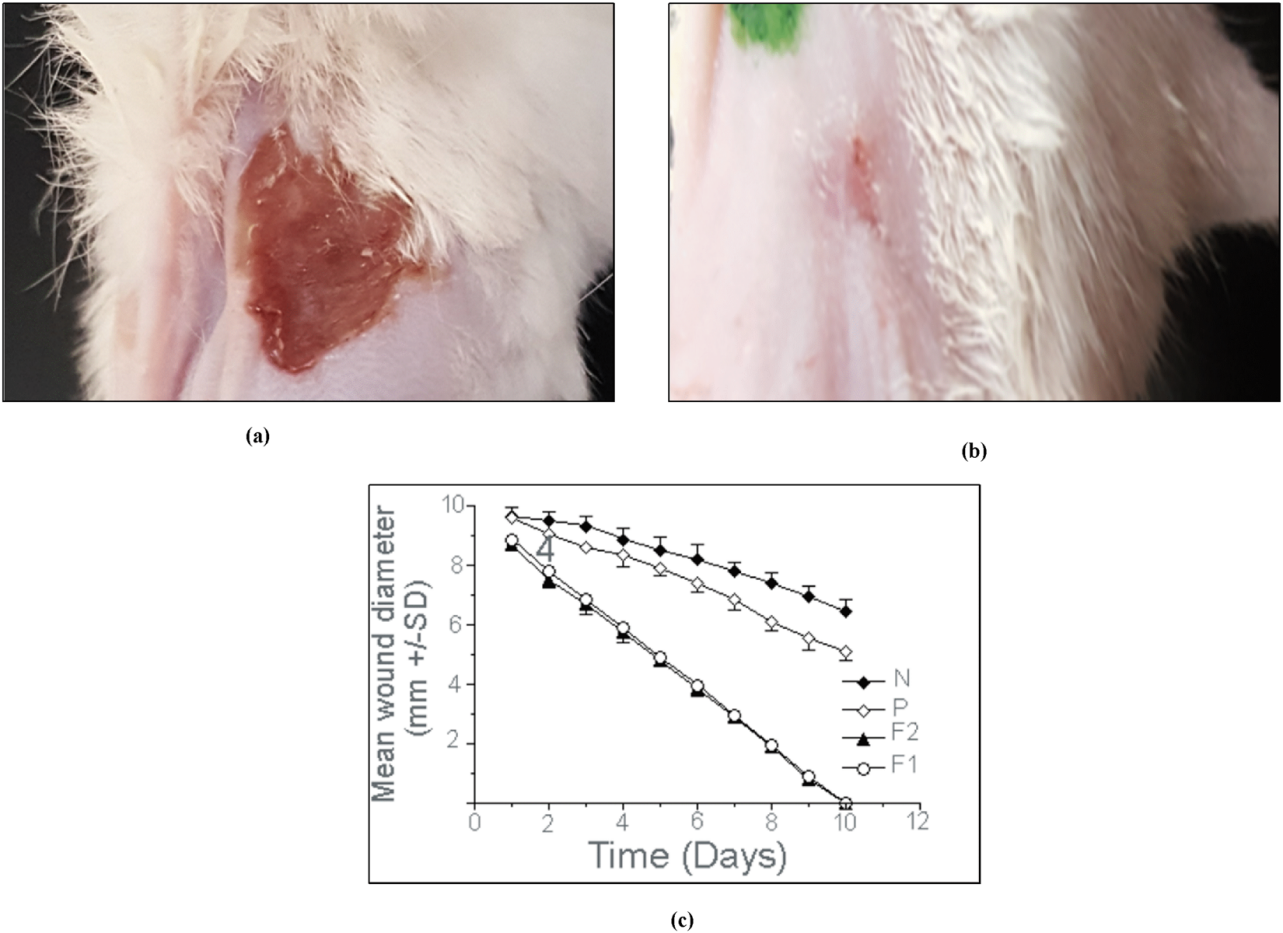
Burn wound position on mouse dorsal skin treated with F1 formula, mouse before application; day 1 (**a**), mouse after end of treatment; day 9 (**b**), mean wound diameter (mm ± SD) daily application (**c**) of F1 (AuNPs-PF127), F2 (AuNPs-PF127-HPMC) and P (silver sulphadiazine cream) compared with N (normal saline).

Both formulations (F1 and F2) were topically administered on burn wound infection sites once daily until complete healing was observed (Fig. 6a,b).

#### The effect of the AuNPs thermoresponsive gels on the inhibition of the wound area

All measurements were take daily throughout the course of treatment. The wounds on all mice that were treated with the prepared formulae, F1 and F2, showed continuous burn edge contraction over the course of treatment. At the end of the treatment period, the maximum burn diameter contraction was reached by both formulae when compared to the commercial product (silver sulphadiazine) (Fig. 6c).

### Bacterial infection assessment of the induced burns

#### *In vivo* antibacterial effects of the AuNPs gels

All swabs from all positions showed reductions in bacterial loads over time. By day 5, no bacteria were detected in the burn sites treated with the tested formula, F1 and F2. In contrast, the burn sites treated with silver sulphadiazine continued to show bacterial growth until day 7 and the saline treated burn sites continued to show superficial bacterial loads until the end of the treatment period (day 9) (Table 4).

**Table 4:** Bacterial loads at different time points after the application of F1 or F2.

Treatment	Time in days									
	0	1	2	3	4	5	6	7	8	9
F1	100	180	−18.7	−83.7	−98.4	<20				
F2	100	186.7	8.7	−67.4	−95.7	<20				
P	100	160	43.3	−25.3	−70.1	−91.9	−99	<20		
N	100	173	91.3	26	−29.3	−72	−91.3	−97.9	−99.8	<20

Bacterial load was calculated as a % of the initial load for each site. F1: Formulation 1; F2.

Formulation 2; P: Positive control, silver sulphadiazine; N: Negative control, saline.

Bacterial load was calculated as a % of the initial load for each site. F1: Formulation 1; F2: Formulation 2; P: Positive control, silver sulphadiazine; N: Negative control, saline.

**Figure 7:**
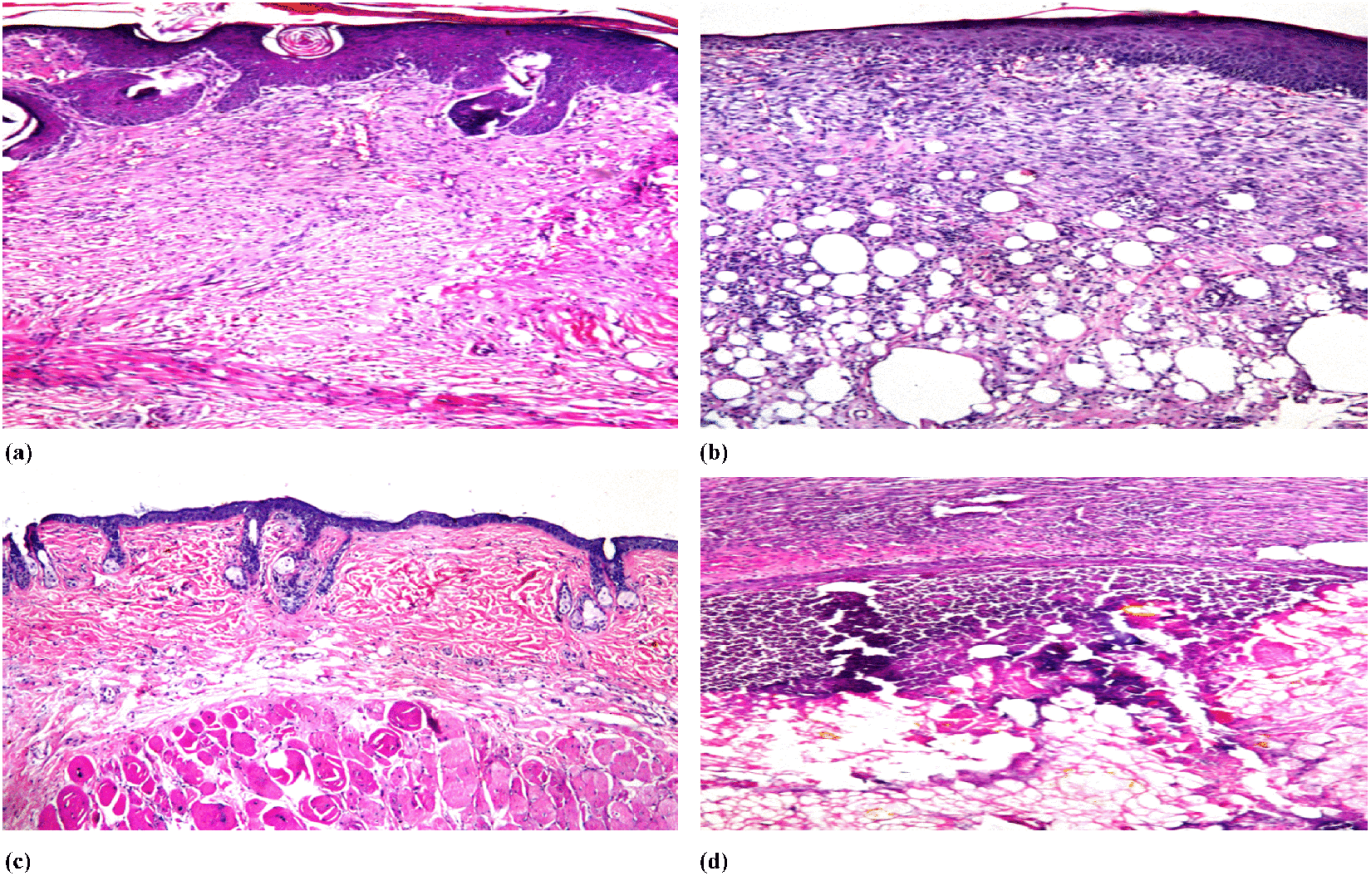
Photomicrographs of tissue sections stained with H and E (16x mag), represent skin of mice treated with silver sulphadiazine, showing focal hyperkeratosis and acanthosis in the epidermis as well as focal granulation tissue formation in the underlying dermis (**a**), skin of mice treated with F2(AuNPs-PF127-HPMC), showing hyperkeratosis and acanthosis in the epidermis with granulation tissue formation and inflammatory cells infiltration in the underlying dermis (**b**), skin of mice treated with F1(AuNPs-PF127), showing intact normal histopathological structure of epidermis and dermis with hyalinosis in underlying skeletal muscle and few inflammatory cells infiltration (**c**) and skin of mice treated with saline showing focal suppuration with liquefaction and pus formation in the subcutaneous tissue (**d**).

Histopathological analysis: The skin autopsy of the control group showed no histopathological alterations, with normal structures of the epidermis, dermis, subcutaneous tissue, adipose and underlying musculature, (Fig. 7) (Table 5).

**Table 5:** The severity of the histopathological alterations observed during skin autopsies of the different experimental groups.

Histopathological alterations	Groups/Score*			
	N	P	F2	F1
Hyperkeratosis and acanthosis in the epidermis	++	++	++	—
Granulation tissue formation in dermis	—	+++	++	—
Inflammatory reactions in subcutaneous tissue	+++	—	++	—
Suppuration in subcutaneous tissue	+++	—	—	—
Hyalinization of skeletal muscle	—	—	+	++
Inflammation in skeletal muscle bundles	—	—	+	+

Score: +++ Severe, ++ Moderate, + Mild, — Nil.

The epidermal layer of the group treated with silver sulphadiazine showed focal hyperkeratosis and acanthosis, while the underlying dermis showed focal granulation tissue formation of the fibroblasts and angioblasts, which formed new blood capillaries, indicating the beginning of the healing process (Fig. 7a).

The group treated with the F2 formula showed focal hyperkeratosis and focal acanthosis in the epidermis, while the underlying dermis showed focal granulation tissue formation with inflammatory cell infiltration, which extended into the subcutaneous tissue. The subcutaneous tissue also showed severe congestion in the blood vessels with focal haemorrhage. There was inflammatory cell infiltration in between the hyalinized skeletal muscles (Fig. 7b).

The epidermis and dermis of the group treated with F1 showed normal intact histological structures, indicating complete healing where no hyperkeratosis or acanthosis could be observed in the epidermis. The underlying dermis did not show any granulation tissue formation and there was no inflammatory reaction in the subcutaneous tissue. Only few inflammatory cells infiltrating in between the hyalinized skeletal muscle could be observed (Fig. 7c). Severe suppuration in the subcutaneous tissue was detected in the group treated with saline, which was not observed in the other groups (Fig. 7d).

Reduction in the wound size:At day nine after application, the positive control (P) produced a significant reduction in wound size of 78.98% of the control (N). Both F1 and F2 produced nearly complete healing of wounds by day nine and wounds were completely healed by day 10 of application. There was no significant difference (p > 0.05) between the effects of F1 and F2. The effects of both F1 and F2 were significant when compared with either P or N.

### Skin irritation and skin sensitization

The potential for F1 to cause skin irritation and sensitization was examined, and it was found that F1 did not produce any undesirable side effects when applied to the skin either once (irritation in rabbits) or repeatedly (sensitization in guinea pigs).

## Discussion

Zeta potentials (ζ) calculate the electrophoretic mobility of AuNPs as a streaming potential surrounding the electric double layer of oscillating electric fields. The negative charge was due to charge stabilization with citrate ions. The small value of the PDI indicated the homogenous distribution of formed nanoparticles. The TEM results of the present study are in agreement with those obtained by Paul *et al*.^54^, who described the AuNPs in their studies as well dispersed, spherical to triangular in shape and 22 nm in size^59^. The samples matched their predicted size within the limit of error and were in accordance with those obtained under particle size investigation.

The gelation temperatures were similar to those reported by Wei *et al*.^60^, who stated that, when PF127 molecules form an aqueous solution, they will arrange themselves to form spherical micelles with an outer shell of hydrated swollen PEO chains that surround the dehydrated PPO core at a concentration and temperature above a critical value. In addition, configuration change may also be used to explain the temperature-dependent gelling of the PF127 solution^61^. PF127 molecules demonstrated a well-arranged zigzag pattern. Upon increasing temperature, a closed packed meander structure was obtained instead of this zigzag form, which resulted in a more glutinous gel. The formation of a stiff gel was due to intermolecular hydrogen bonding and the consequent development of a network between the free hydroxyl groups of HPMC and PF127 molecules. Furthermore, the low gelling temperature of the PF127-HPMC mixture was due to the formation of a hydrogen bond between an oxygen atom of PF127 and a proton of water, in addition to hydrogen bonding that existed after the addition of HPMC to the system, resulting in the formation of a stiff gel that requires less heat energy^62,63^. The total gelling process of PF127-HPMC was typically divided into two steps. The first step occurs when the temperature increases to reach the critical micelle temperature that results in the aggregation of PF127-HPMC monomers to form spherical micelles. In the second step, a further increase in the temperature packs the micelles in an orderly manner to form gels^64,65^. Temperature, polymer concentration and additives have a tremendous effect on gel strength, which has a direct impact on the sustained release pattern of a drug. These results can be explained by Prudhomme *et al*.^66^, who reported that a gel was formed when a suitable concentration of PF127 polymer molecules were mixed with water to form a solution in which the spherical micelles of the polymer were arranged as the simple network structure of gel. When the volume of micelles was low, a region with low micelle concentration was created where micelles were separated, resulting in an isotropic Newtonian fluid. Next, micelles were affected by temperature, which caused the hydration of ethylene oxide units. Consequently, the Newtonian fluids converted into non-Newtonian fluids that form a gel. As stated earlier, upon raising the temperature of the thermoreversible PF127 and HPMC formulae, the gel strengths increased and they formed gels^63^. This result can be explained by the decomposition of the hydrogen bonding found between the polymer blocks, resulting in the aggregation of polymer molecules into micelles. Increasing temperatures cause the sizes and numbers of micelles to increase, causing the PF127 solution to turn into a stiff gel^62^. As mentioned previously, the addition of HPMC favoured dehydration and, therefore, gelation occurred at relatively lower temperature.

The increase in bio-adhesive force may be due to the arrangement of the gel molecules in a lattice pattern. Furthermore, the molecular weight of HPMC also affected the bio-adhesive force, as the addition of the high molecular weight HPMC to the gel increased its viscosity and strength, which, in turn, increased its bio-adhesive force.

The formulation consisting of PF127 and HPMC exhibited a higher viscosity than that containing only PF127, as HPMC is characterized by a high number of OH groups that permit HPMC to endure physical cross-linking with PF127 through its PPO chains. After dehydration occurred, the adjacent molecules became more closely packed. Spherical micelles were obtained, resulting in an increase in the intrinsic viscosity. At 30 °C and higher temperatures, approximately all of the polymer was found in the micelle form and dehydration of the micelle shell was reduced, resulting in the dominating factor being the intrinsic viscosity of the micelle^67^. The attributes of *in vitro* release were due to the addition of HPMC to the PF127 gel, which reduced the release of AuNPs and allowed the formulation to remain at the target site^38^.

When HPMC is mixed with water, three-dimensional physical networks are formed, resulting in a firmly oriented gel buildup. Thus, HPMC hindered the dissolution of gel, as expected. This led to a delay in the drug diffusion and consequently controlled and sustained the release rate of AuNPs.

The *ex vivo* permeation results obtained in the present study were in agreement with the results obtained by Katiyar *et al*.^68^, who reported the low permeation of AuNPs from thermoresponsive gels within 2 h, indicating a sustained release profile.

The kinetic results may be due to the continuous swelling-controlled behaviour of the PF127 system, combined with the instant dissolution of the polymer. Furthermore, the formulation dissolution behaviour that is exhibited by swelling-controlled release systems may explain the process of drug release. This new theory allows for a better understanding of polymer dissolution^69^, in which drug–polymer–solvent systems exhibit drug release patterns that may be dissolution-controlled or diffusion-controlled. In the current experiments, a dissolution-controlled release mechanism can explain the zero-order release of AuNPs from PF127.

A successful antibacterial agent should be able to decrease the bacterial load (bioburden) in wounds and, consequently, prevent infection. Skin burns and wounds, being the most exposed tissues, are particularly susceptible to bacterial contamination and infections^70–72^. It is estimated that mortality from burn wound infections may approach or exceed 50%, even with aggressive antibiotic treatment^73^.

Wound and burn bacteria can frequently colonize and proliferate in the exposed tissues and, when established, these bacteria can pass through the blood capillaries of the tissues, which may eventually lead to bacteraemia. Furthermore, these bacteria may result in endotoxic shock due to the release of virulence factors. Once the bacteraemia and/or endotoxic shock phases are reached, infections from burns or wounds become untreatable by traditional antibiotic treatments. This makes it imperative that antibiotic therapy be administered at an early stage of infection to be effective.

Gold, similar to silver, when used in reasonable quantities, has no negative effects on the human body^74^. Several experimental studies on the multiple applications of AuNPs as therapeutic and diagnostic agents in medicine have been reported. One of the most interesting applications of AuNPs is as novel transdermal drug delivery systems. R. Gupta *et al*.^75^ studied the permeation of AuNPs through human and rat skin using thiol coated, neutral, hydrophobic, cationic and anionic AuNPs with skin lipid membranes and concluded that AuNPs can penetrate the human skin as a novel transdermal drug delivery system.

Z. Jiménez-Pérez *et al*.^76^ studied the multifunctional properties and effects of AuNPs as bioactive agents with no toxic effects to human dermal layers. K. Niska *et al*.^77^ showed a formation of gold nanoparticles that offers a new approach for introducing new therapeutic options to dermatological infectious and skin diseases, with high biocompatibility and decreased toxicity, thus, improving the effectiveness and safety of therapy to human skin cells. G. Sonavane *et al*.^78^ demonstrated AuNPs permeation through mouse skin using a TEM study of rat skin that revealed the accumulation of smaller size AuNPs in deeper regions of the skin, whereas larger particles were observed primarily in the epidermis and dermis. The presence of gold inside the rat skin was confirmed by EDS. They concluded that AuNPs would be an interesting carrier for transdermal delivery.

C. J. Murphy *et al*.^79^ investigated the degree of AuNPs toxicity to human cells, and the obtained data confirmed that none of the spherical gold nanoparticles were toxic to the human cells. In the same study, similar viability studies were performed using immune system cells that showed that gold nanoparticles were not cytotoxic and that they reduced the amount of potentially harmful reactive oxygen species in the cells. In addition, citrate-capped gold nanospheres were not toxic according to an assay in skin cells.

H. Bessar *et al*.^80^ prepared a novel AuNPs methotrexate conjugate and concluded that it is an effective non-toxic carrier for the satisfactory percutaneous absorption of methotrexate, which could help in the possible topical treatment of psoriasis. F. Larese Filon *et al*.^81^ reported that AuNPs can easily permeate the skin, which is an important property for biomedical applications, and suggested that this type of NPs might be a carrier for transdermal delivery because it is able to penetrate the human skin with no toxic effect. J. Lee *et al*.^82^ successfully produced biocompatible AuNPs used in skin recovery and wound healing through a transdermal delivery method.

S. Gupta *et al*.^83^ reported that AuNPs, in particular, are an excellent intracellular targeting vector used for transdermal delivery of substances, due to their biocompatibility, deeper targeting of cutaneous tumours, sustained release, low toxicity and drug adhesion to the skin. W. Gao *et al*.^84^ studied the slow release of AuNPs from a gel, which showed an enhanced efficacy in treating bacterial infections and skin inflammation without significant cytotoxicity. On the contrary, this system was used to remove toxic compounds from the body.

E. A. Sykes *et al*.^85^ reported that AuNPs, used in diagnostics, were selected to be detected in mouse skin, where neither localized nor systemic toxicity was observed. S. Kalita *et al*.^86^ reported that AuNPs are biocompatible towards mammalian cells and have antibacterial properties. As metallic nanoparticles, they are used in small doses because of their controlled release effect, thereby diminish any proposed cytotoxicity by reducing the necessity for high doses.

The avoidance of cytotoxicity explains the reasoning for choosing the 0.5 µg AuNPs concentration in the present study, which represented the minimum effective concentration, as it showed an identical level of antibacterial activity when compared with the higher dose.

Of the two prepared formulae, F1 showed rapid *in vitro* antibacterial activity when compared to F2, according to measurements of the zones of inhibition after 24 h, and F1 showed a higher release rate than F2. However, F2 demonstrated a sustained pattern, which is in agreement with Parhi^32^, who stated that polymers that enhance gel strength and bio-adhesive force while decreasing the gelation temperature could distort or squeeze the diffusion channels, delaying the release process, as observed for F2, in contrast with F1, where the diffusion of the drug was higher. In addition, the induced burn associated with infection resulted in an increase in the temperature at the infected site, which led to an increase in the gel strength and viscosity of the thermoresponsive gel F2, which could delay but sustain its healing effect. In contrast, F1, being less viscous, possessed less gel strength, which enhanced its effect compared to F2.

To determine the *in vivo* antibacterial efficiency of the prepared AuNPs (F1 and F2), both formulae were topically applied on a mouse model with induced burn wounds infected with *Staphylococcus aureus*. *Staphylococcus aureus* was chosen as a test strain for evaluating the antibacterial activity of AuNPs because this is the most abundant strain identified in infected burn wounds^87^. Bacterial load results from all positions showed a gradual decrease over time. F1 and F2 results were comparable, where no bacterial growth was detected by day 5 of treatment.

AuNPs disturb the normal functioning of the bacterial cell wall and cytoplasmic proteins responsible for the cell function, finally causing cell death^88^. There are several suggested mechanisms for the antibacterial activity of AuNPs, including that they may change the membrane potential and reduce adenosine triphosphate (ATP) synthase actions, thus decreasing the metabolic processes. In addition, AuNPs cause a decline in the subunit of the ribosome for tRNA binding, consequently collapsing its biological mechanism. AuNPs are small sized, which enhances the surface area, leading to the production of some electronic effects that are useful for enhancing the surface reactivity of NPs. This relatively high surface area provides enhanced contact with bacteria. These significant features greatly enhance the antibacterial activity of the AuNPs. Other mechanisms suggest the disruption of the bacterial respiratory chains due to the binding of AuNPs to the thiol groups of enzymes, such as nicotinamide adenine dinucleotide (NADH) dehydrogenases, resulting in the discharge of oxygen species, leading to oxidative stress. Consequently, significant destruction occurs in the bacterial cell structure, which leads to cell death.

F1 and F2, applied topically to mouse skin, were found to be very effective for the local antibacterial treatment of burn wound infections, resulting in the apparent complete skin healing compared to the positive control group treated with silver sulphadiazine or the negative saline control group. This was in accordance with the results obtained from the *ex vivo* permeation study. The increases in the gel strength, viscosity and bio-adhesive force of the polymer appeared to be involved in the retarded release and diffusion of the drug, which was evident by the histopathological study, where the group treated by the F2 formula showed moderate hyperkeratosis and acanthosis in the epidermis, accompanied with granulation tissue formation in dermis and inflammatory reactions in the subcutaneous tissue, while the group treated with F1 did not show any of these histopathological alterations.

Swabs from normal saline (0.9% NaCl) positions showed a superficial bacterial load reduction over time, as mentioned in the literature^89^, while the histopathological results of the skin biopsies of the infected burn sites treated with saline showed severe suppuration in the subcutaneous tissue. This result may be attributed to the lack of any deep clearance of the bacteria during saline application or biofilm formation. This was not the case with other treatments, where no suppuration was detected in the underlying skin layers, indicating the deep clearance of bacteria exerted by the high antibacterial activity of the applied treatments and the excellent penetration through underlying tissue.

## Conclusion

Overall, the results obtained in the present study have shown that the developed thermoresponsive gels are promising candidates as carrying vehicles for AuNPs formulations that exhibited more prolonged and sustained effects compared to a AuNP suspension. *In vitro*, *ex vivo* and *in vivo* investigations showed the improved bioavailability, skin permeation, antibacterial and anti-inflammatory activity of the prepared AuNPs thermoresponsive gels. Furthermore, AuNPs thermoresponsive gels did not show any signs of skin irritation in rabbits or sensitization in guinea pigs, which offer AuNPs-PF127 and AuNPs-PF127-HPMC gels as smart topical antibacterial drug delivery systems in cases of skin inflammation and wound healing.
